# Functional interplay between glutathione and hydrogen sulfide in regulation of thiol cascade during arsenate tolerance of common bean (*Phaseolus vulgaris* L.) genotypes

**DOI:** 10.1007/s13205-015-0285-6

**Published:** 2015-03-04

**Authors:** Dibyendu Talukdar

**Affiliations:** Department of Botany, R.P.M. College (University of Calcutta), Uttarpara, West Bengal 712258 India

**Keywords:** Arsenate, BSO, Thiol cascade, Sulfide, Ascorbate, *Phaseolus vulgaris*

## Abstract

Changes in expressions of up- and downstream thiol cascade were studied in leaves of *Phaseolus vulgaris* L. cv. VL-63 and its mutant, *pvsod1* (deficient in superoxide dismutase activity) under 50 μM sodium arsenate (As), As + l-buthionine-sulfoximine (BSO) and As + BSO + Sodium hydrosulfide (NaHS)-treatments for 10 days. Main objective was to investigate the functional relationship between hydrogen sulfide (H_2_S) and glutathione (GSH) in regulation of sulfate transporters and cysteine metabolisms as up-stream thiol components and GSH, phytochelatins (PCs) and antioxidant defense response as downstream cascade under As-exposure. As treatment alone initiated coordinated inductions of sulfate transport, biosynthesis of cysteine, GSH, and PCs, and GSH-mediated antioxidant defense in the *pvsod1* mutant. At As + BSO, GSH synthesis was blocked, resulting in significantly low GSH redox pool and steep decline in GSH-dependent antioxidant capacity of both the genotypes. However, unlike VL-63, cysteine-degradation pathway was induced in *pvsod1* mutant, resulting in significant accumulation of endogenous H_2_S. The H_2_S-surge in the *pvsod1* mutant stimulated ascorbate-dependent antioxidant defense and catalases and regulated O-acetylserine (thiol)lyase activity, preventing overaccumulation of H_2_O_2_ and free cysteine, respectively. No As-induced oxidative stress symptom was observed in the mutant. This trend was maintained at As + BSO + NaHS treatment, also. In contrast, failure to induce entire cascade from sulfate transport to downstream antioxidant defense led to onset of As-induced oxidative damage in VL-63 plant. Results revealed dual roles of H_2_S as (a) stimulator of GSH-independent antioxidant defense and (b) regulator of cysteine homeostasis through its metabolic diversion during As-exposure and blockage of GSH biosynthesis.

## Introduction

Arsenic (As) is a ubiquitous toxic and carcinogenic metalloid. Food crops such as rice, pulses and vegetables grown in As-contaminated soil can accumulate high levels of As (Bhattacharya et al. 2010). Common bean (*Phaseolus* *vulgaris* L.) is a widely grown antioxidant-rich food legume (Liao et al. 2012) but the crop is sensitive to As (Stoeva et al. 2005; Talukdar 2013). Being grown in aerobic fields, legumes are usually exposed to arsenate (AsV) form of As which either directly or through conversion to highly toxic arsenite (AsIII) adversely affects plant growth by generating excess reactive oxygen species (ROS) and consequent oxidative damage to membrane structure and function (Gupta et al. 2008).

Sulfur (S) is an essential nutrient for plant growth and development, and is generally taken up by plants in the form of sulfate through dedicated sulfate transporters (Sultr) (Kopriva et al. 2012). Sulfate is then reduced to sulfide and ultimately incorporated into the amino acid skeleton of O-acetylserine (OAS) by the O-acetylserine (thiol) lyase (OAS-TL) (Hell and Wirtz 2011; Takahashi et al. 2011). Cysteine (Cys) is the first committed molecule in plant metabolism that contains both S and nitrogen, and, thus, its metabolic regulation is of utmost importance for the synthesis of a number of essential metabolites in plant pathways (Kopriva et al. 2012). Conglomerations of thiol transport as well as assimilation and thiol-dependent antioxidant defense comprise thiol cascade and thiol-ligand glutathione (GSH) play central roles in this cascade (Finnegan and Chen 2012). Growing evidences suggest that decreased activity of Cys-synthesizing machinery ultimately compromises GSH and phytochelatin (PC) synthesis and effectiveness of antioxidant defense in As-sensitive plants (Srivastava et al. 2009). GSH synthesis is catalyzed by γ-glutamylcysteine synthetase (γ-ECS) in a rate-limiting way (Noctor et al. 2012). Besides formation of PCs, GSH is an integral part of ascorbate (AsA)-GSH antioxidant cycle in which dehydroascorbate reductase (DHAR), ascorbate peroxidase (APX) and glutathione reductase (GR) along with catalases (CAT) and GSH-S-transferase (GST) outside the cycle play pivotal roles in redox homeostasis (Noctor et al. 2012). While sulfur uptake/transport and Cys/GSH synthesis are the key components at the up-stream, GSH-dependent antioxidant defense constitute the downstream thiol cascade during plants’ response to stress.

Knowledge regarding metabolic channeling of Cys into different routes to regulate plant growth and development has recently been widened with the discovery of role of Cys-generated hydrogen sulfide (H_2_S) as a prominent signaling molecule in plants (Calderwood and Kopriva 2014). Recent research has confirmed that most of the endogenously synthesized H_2_S occurred through the desulfuration of l-Cys and d-Cys by l-cysteine desulfhydrase (LCD) and d-cysteine desulfhydrase (DCD), respectively (Álvarez et al. 2010). H_2_S participates in diverse physiological activities to promote plant growth processes and abiotic stress tolerance (Chen et al. 2011; Calderwood and Kopriva 2014). However, it is still not clear whether these effects are from H_2_S alone or these are mediated by downstream thiol-antioxidant metabolites like GSH.

Substantial progress has been made in revealing central roles of GSH during As-detoxification. However, it is not known whether Cys degradation and endogenous H_2_S has any roles in mitigating As-toxicity in crop plants. Recently, an EMS-induced bean mutant *pvsod1* deficient in SOD activity was found tolerant to As (V) stress. Primary investigation revealed possible interplay between GSH and Cys-degraded H_2_S in mitigating As-stress in the mutant. Main objectives of this study were therefore to explore (1) the response of sulfate transport and its assimilation into Cys, (2) the roles of GSH and response of antioxidant defense enzymes, and (3) involvement of Cys metabolisms other than GSH in leaves of common bean genotype subjected to As (V) treatment. The study hypothesized that functional interplay exists between two Cys-metabolites, the GSH and H_2_S, during As-induced oxidative stress in leaves of common bean.

## Materials and methods

### Plant material, growth conditions and treatment protocol

Fresh seeds of common bean (*Phaseolus vulgaris* L.) cultivar VL-63 and one *pvsod1* mutant exhibiting low superoxide dismutase (SOD) activity (25 % of wild type) (Talukdar and Talukdar 2013) were surface sterilized with NaOCl (0.1 %, w/v) and continuously washed under running tap water followed by distilled water. Seeds were allowed to germinate in the dark in two separate sets on moistened filter paper at 25 °C. Germinated seedlings were randomly placed in polythene pots (10 cm diameter and 12 cm high, 10 plants pots^−1^) containing 250 ml of Hoagland’s No 2 nutrient media, and were allowed to grow for 10 days. Seedlings of both the genotypes were then subjected to (a) 50 μM of As (sodium arsenate, MW 312.01 g mol^−1^; technical grade, purity 98.5 %, Sigma-Aldrich, Bangalore, India) treatment, (b) As + 1 mM BSO (l-buthionine-sulfoximine, Sigma-Aldrich, Bangalore, India), (c) As + 1 mM BSO + 100 μM NaHS (Sodium hydrosulfide, SRL, Mumbai, India) and were allowed to grow for another 10 days. BSO was used as specific inhibitor of GSH biosynthesis, while NaHS was the exogenous H_2_S donor. Pilot experiments indicated significant effect of arsenate, BSO and NaHS at these concentrations on plant biomass (Talukdar 2013; Talukdar and Talukdar 2013) and thus, were selected for the present study. Untreated plants were used as control; cultivar as mother control (MC) and mutant line as mutant control (MuC). The experiment was carried out in a completely randomized block design in an environmentally controlled growing chamber under a 14-h photoperiod, 28/18 (±2 °C), relative humidity of 70 ± 2 %, and a photon flux density of 150 μmol m^−2^ s^−1^. Nutrient solution was refreshed thrice per week, and all experiments were conducted thrice with four replicates. After 10 days, As-exposed seedlings were harvested along with control, carefully washed with distilled water, blotted gently, and were oven-dried at 60 °C till constant weight. Root and shoot dry weights were measured and fresh leaves were used for metabolic and molecular analysis.

### Determination of As content and endogenous H_2_S

As concentration in dried root and shoot samples was measured by digestion methods (HNO_3_–HClO_4_ mixture at 3:1, v/v) using flow injection-hydride generation atomic absorption spectrophotometer (Perkin-Elmer, FIA-HAAS Analyst 400) and keeping Standard Reference Materials of tomato leaves (item number 1573a, from National Institute of Standards and Technology, USA) for part of the quality assurance/quality control protocol, as detailed earlier (Talukdar 2013). The translocation factor (TF) is the ratio of the level of As in shoots upon roots. Endogenous H_2_S was determined by the formation of methylene blue from dimethyl-p phenylenediamine in H_2_SO_4_ following Sekiya et al. (1982) and Chen et al. (2011).

### Measurement of glutathione, ascorbate, Cys and assay of thiol-metabolizing enzymes

Reduced and oxidized form of ascorbate and glutathione were measured following the methods of Law et al. (1983) and Griffith (1980), respectively. For enzyme assay, plant tissue was homogenized in buffers specific for each enzyme under chilled conditions. The homogenate was squeezed through four layers of cheese cloth and centrifuged at 12,000×*g* for 15 min at 4 °C. The protein content of the supernatant was measured following Bradford (1976) using BSA as standard. The OAS-TL (EC 2.5.1.47) activity was assayed by measuring the production of l-Cys (Saito et al. 1994). Cys content was measured spectrophotometrically (Perkin-Elmer, Lambda 35, Mumbai, India) at 560 nm following Gaitonde (1967). Assay of γ-ECS (EC 6.3.2.2), PC synthase (PCS; EC 2.3.2.15) and LCD (EC 4.4.1.1) was done by following Seelig and Meister (1984), Howden et al. (1995), and Bloem et al. (2004), respectively. DCD (EC 4.4.1.15) activity was determined in the same way, but d-Cys was used instead of l-Cys (Riemenschneider et al. 2005).

### Assay of antioxidant enzymes and glycolate oxidase (GO)

Leaf tissue of 250 mg was homogenized in 1 ml of 50 mM K-phosphate buffer (pH 7.8) containing 1 mM EDTA, 1 mM DTT, and 2 % (w/v) polyvinyl pyrrolidone using a chilled mortar and pestle kept in an ice bath. The homogenate was centrifuged at 15,000×*g* at 4 °C for 20 min. Clear supernatant was used for enzyme assays. Soluble protein content was determined using BSA as a standard (Bradford 1976). SOD (EC 1.15.1.1) activity was determined by nitro blue tetrazolium (NBT) photochemical assay (Beyer and Fridovich 1987) and expressed as unit per minute per milligram protein. One unit of SOD was equal to that amount causing a 50 % decrease of SOD-inhibited NBT reduction. APX (EC 1.11.1.11) activity (nmol AsA oxidized min^−1^ mg^−1^ protein) was assayed following Nakano and Asada (1981) with H_2_O_2_-dependent oxidation of AsA followed by a decrease in the absorbance at 290 nm (*ε* = 2.8 mM^−1^ cm^−1^). DHAR (EC 1.8.5.1) and GR (EC 1.6.4.2) activity was measured following the protocol of Nakano and Asada (1981) and Carlberg and Mannervik (1985), respectively. CAT (EC 1.11.1.6) extraction was performed in a 50-mM Tris–HCl buffer. The enzyme activity was assayed by measuring the reduction of H_2_O_2_ at 240 nm (*ε* = 39.4 mM^−1^ cm^−1^) and 25 °C, as detailed earlier (Talukdar 2013). GSTs (EC 2.5.1.18) specific activity was assayed following Li et al. (1995). GO (EC 1.1.3.15) activity was assayed by the formation of a glyoxylate-phenylhydrazone complex at 324 nm (Baker and Tolbert 1966) and was expressed as µmol glyoxylate min^−1^ mg^−1^ protein.

### Estimation of foliar H_2_O_2_ content, lipid peroxidation and electrolyte leakage (EL) %

Leaf H_2_O_2_ content and membrane lipid peroxidation rate were determined following Wang et al. (2007) and by measuring the malondialdehyde (MDA) equivalents (Hodges et al. 1999), respectively. Electrolyte leakage (EL %) was measured according to Dionisio-Sese and Tobita (1998).

### Relative gene expression analysis through quantitative RT-PCR

Total RNA isolation and first-strand cDNA synthesis were done following manufacturer’s (Chromous Biotech, Bangalore, India) instructions, as detailed earlier (Talukdar and Talukdar 2014a). The quality of total RNA samples was determined spectrophotometrically (Systonic, Kolkata, India) from *A*260/280 ratio as well as by 1 % agarose gel electrophoresis, and 600 ng of mRNA was used for cDNA synthesis. Quantitative RT-PCR of first-strand cDNA was run on ABI Step-One (Applied Biosystems, Foster City, CA, USA) Real-Time PCR machine. Amplification was done in a total reaction volume of 50 μl, containing template (first-strand cDNA) 2.0 μl, forward and reverse primer 2.0 μl each with 50 nM μl^−1^ concentration, 2 × PCR SYBR green ready mixture (Fast Q-PCR Master Mix, Chromous Biotech, India, cat no. QCR 05/QCR 06), 25.0 μl, and DEPC water 19.0 μl. Primers for selected genes were constructed by Primer Express™ V. 3.0 software (Applied Biosystems, USA) with the search of available sequence databases (http://www.phytozome.net/commonbean.php; http://plantgrn.noble.org/PvGEA/) and reports on *Phaseolus vulgaris* (Liao et al. 2012; Talukdar and Talukdar 2013) and were presented in Table 1. The qRT-PCR cycling stages comprised of initial denaturation step at 94 °C (3 min), followed by 35 cycles of 94 °C (5 s), 62 °C (10 s), 72 °C (10 s) and a final extension stage at 72 °C (2 min). A melting curve analysis was performed after every PCR reaction to confirm the accuracy of each amplified product. Samples for qRT-PCR were run in four biological replicates with each biological replicate contained the average of three technical replicates. RT-PCR reaction mixtures were loaded onto 2 % agarose gels in TAE buffer. A 100-bp DNA ladder was run on every gel. The mRNA levels were normalized against a common bean *ubiquitin* as the housekeeping gene, and the relative (fold change to control value) expression of target genes was calculated as 2^−ΔΔCt^ (Livak and Schmittgen 2001).

**Table 1:** Oligonucleotide primer sequence (5′ → 3′) used in qRT-PCR reactions

Candidate genes	Forward primers	Reverse primers
*PvSultr1;1*	CGTTCGTCAGAGAGTGCTAGCTCC	ATGTGTTTATGTATATGAATAGAC
*PvSultr1;2*	ATCGGTGGACATGTATCCGATGA	CATGTGTAGCTTGCCTATCACCAA
*PvSultr2;1*	GATCATAGTTCAAACTTCCACACA	CCGTAAATTCATCACATGCAATAA
*PvSultr2;2*	CTTGGATCCATGGCATAGAGCTTC	GTATCCATCAACACAACTCGGGGA
*OAS*-*TLA*	CGGCACAAGATTCAAGGGATAA	ATCATGGCTTCCGCTTCTTTG
*OAS*-*TLB*	AAACAGCGACGTCGTTTTGCAGCT	CTCTTCTCGAATCGACTGGAAAAG
*γ*-*ECS*	AGCTGTGTCCCACCGAGTG	AAGAGTGAGCGGAGGAGGGT
*PCS*	CCTAATGGAATCTGATGTGCCTT	CTTCTTTGACAGTCGACGAGCCTT
*PvLCD*	GATGCAATGTATTTGTCTCTTTTTC	TTTCTTTTTATAATCTTTTGCTCCC
*PvDCD*	AGAACAGTTCTCTCTTTTTGTCGA	GGACAGTCCACCTTAGAGGCTAGA
*GO*	TAGTTCTCGTGCTGTTGCCGATA	AAGGATATGCTGTTACATTACGTT
*Cu/ZnSODI*	GGCTGTATGTCAACTGGACCTCATTTCA	TGTCAACAATGTTGATAGCAGCGG
*Cu/ZnSODII*	GGATATATGGCATCTGTAACTCATATGC	GCATAAGAATGCTGATAGACAGGG
*MnSOD*	AGTCAAGTTGCAGAGTGCAATCAAGTTC	CAAAGTGATTGTCAATAGCCCAAC
*FeSOD*	AACAAGCAAATAGCCGGAACAGAACAC	AGAAATCGTGATTCCAGACCTGA
*APX I*	CACTTGGCCCTGGACCGTTGTTGTT	CCAGAACCGTCCTTGTAAGTTGC
*APX II*	CAGAGGAGAGTGAAGGCAAAGC	GTCAGTCAAGCTGCATACGATA
*APX III*	GCGACTTCTCCAGCCGATCAAATC	AGGACATTGGTCAGGTCCAG
*DHAR*	CCTAACAAACCCGAATGGTA	ACGGGCACCTTTCCTTCAG
*GRI*	GAAATTGCTAGTCTGTATGCGTCA	AGCAAACTCCAAGGCACAATGT
*GRII*	GCACTGCTCTTCACGTAGACCGCT	AATGGCTGTGGGTGATGTCCGAA
*CAT*	TACTCAGAGGCACCGTCTTG	CTCCTCATCTCGGTGCATAA
*Ubiquitin*	GCTTCGTGGTGGAATGCAGAT	TCGCACCTTGGCAGACTACAA

### Statistical analysis

Data are mean ± standard error (SE) of at least four replicates. Simple *t* test was performed to assess significant differences (*P*_two tailed_) between control and treated values. Variance analysis was performed on all experimental data, and statistical significance (*P* < 0.05) of means was determined by Duncan’s multiple range test using SPSS software (SPS Inc., USA v. 10.0).

## Results

### Changes in plant dry weight and As accumulation

**Fig. 1:**
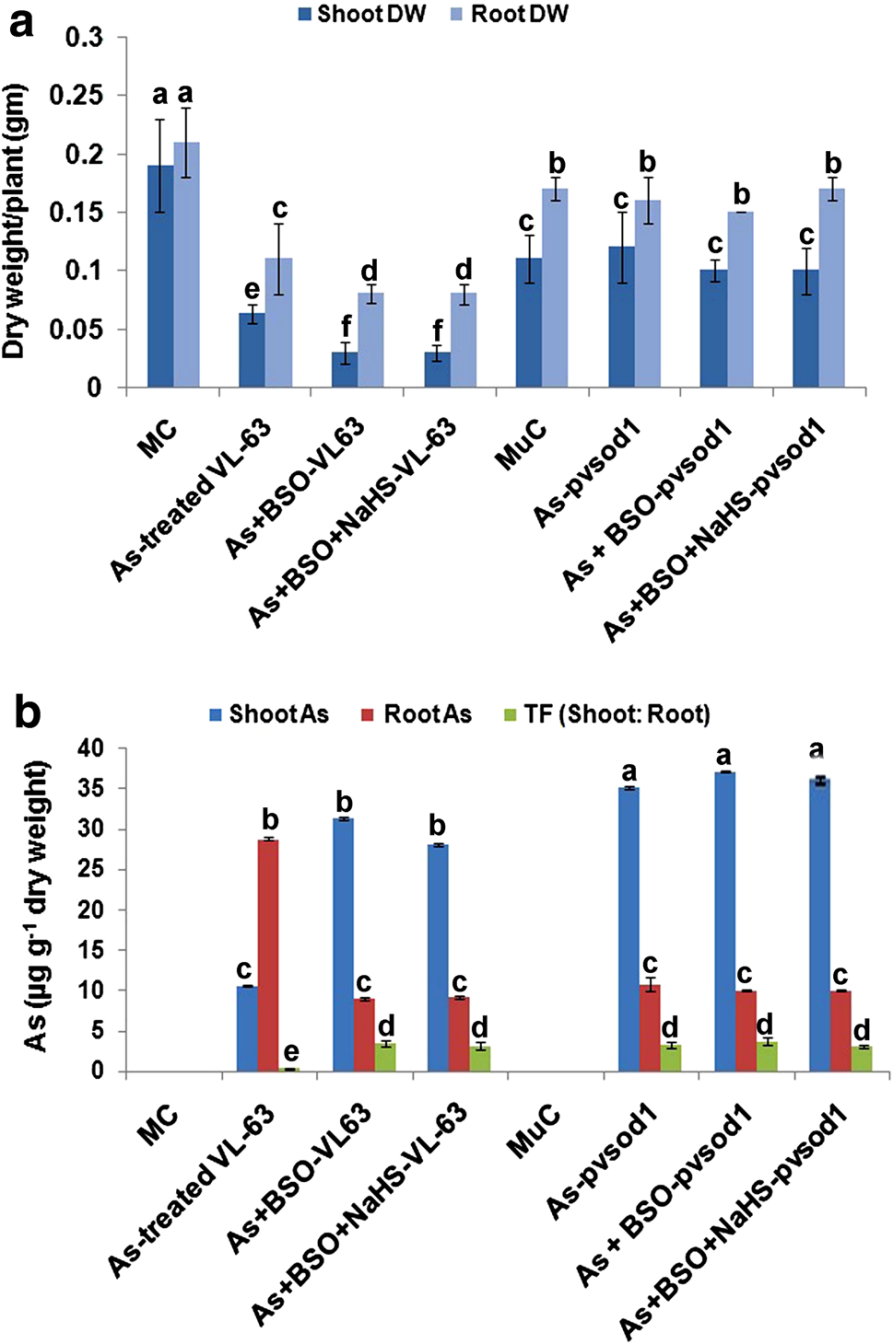
Changes in **a** shoot and root dry weight (g) and **b** as accumulation and transfer (TF) ratio in *Phaseolus vulgaris* L. mother genotype VL-63 and its mutant *pvsod1* in control (*MC* mother control, *MuC* mutant control, 0 μM of As), As (Sodium arsenate, 50 μM)-treated, As + 1 mM BSO, and As + 1 mM BSO + 100 μM NaHS treatments. Data are mean ± SE of four replicates of three independent experiments. Means followed by *the different lowercase letters* were significantly different at *P* < 0.05 using ANOVA followed by Duncan’s multiple range tests

Both shoot and root dry weight in VL-63 seedlings reduced significantly (*P* ≤ 0.0001) in comparison to MC at 50 µM As, further reduced at As (50 µM) + 1 mM BSO, and did not change significantly (*P* = 0.483) at As + 1 mM BSO + 100 μM NaHS in relation to As + BSO. Dry weight of *pvsod1* mutant compared to MuC did not change significantly in any of the three treatment protocols (Fig. 1a). At 50 µM, As accumulation was markedly higher in roots than that of shoots (*P* ≤ 0.0001) in VL-63 (Shoot/root TF < 1) while completely opposite scenario was observed in the *pvsod1* mutants (Fig. 1b). As concentration in shoot and root of the VL-63 turned reverse (Shoot/root TF ratio > 1; *P* ≤ 0.0001) to the above under As + BSO and As + BSO + NaHS. Significantly (*P* ≤ 0.0001) higher As level than the VL-63 was estimated in shoots of *pvsod1* mutant (TF > 3.0) throughout the treatment regimes (Fig. 1b).

### Foliar GSH and AsA content and their redox states

Total GSH and AsA content and their corresponding redox states increased significantly (*P* = 0.025–0.010) over MuC in leaves of the As-treated *pvsod1* mutant. GSH and AsA level reduced while their oxidized forms increased substantially in VL-63 exposed to As + BSO and As + BSO + NaHS (Table 2). In *pvsod1* mutant, GSH redox declined nearly fivefold but AsA redox increased significantly over MuC in the latter two protocols (Table 2). Change was non-significant in rest of the cases.

**Table 2:** Foliar GSH and GSSG (nmol g^−1^ FW), GSH redox [GSH/(GSH + GSSG)], AsA and DHA (nmolg^−1^ FW), AsA redox [AsA/(AsA + DHA)], Cysteine (Cys, nmol g^−1^ FW) and H_2_S (µmol g^−1^ FW) in mother genotype (*Phaseolus vulgaris* L.) VL-63 and the *pvsod1* mutant in 50 µM As (sodium arsenate), As + 1 mM BSO and As + BSO + 100 μM NaHS for 10 days treatment period

Traits	MC (VL-63)	MuC (*pvsod1*)	As-treated		As + BSO		As + BSO + NaHS	
			VL-63	*pvsod1*	VL-63	*pvsod1*	VL-63	*pvsod1*
GSH	161.3 ± 3.8b	130.5 ± 4.8c	157.3 ± 3.5b	220.8 ± 4.3a	76.5 ± 4.7e	59.7 ± 3.2e	86.5 ± 4.7d	53.7 ± 3.2e
GSSG	18.8 ± 1.3c	11.1 ± 0.91c	19.7 ± 1.4c	30.1 ± 1.2b	101.1 ± 5.1a	104.3 ± 4.7a	90.1 ± 5.1a	109.3 ± 4.7a
GSH redox	0.901 ± 0.07a	0.922 ± 0.07a	0.890 ± 0.08a	0.880 ± 0.11a	0.431 ± 0.07b	0.364 ± 0.03c	0.487 ± 0.05b	0.330 ± 0.03c
AsA	800.8 ± 7.1a	690.8 ± 6.8b	793.8 ± 6.8a	779.1 ± 7.2a	332.9 ± 4.1c	840.7 ± 7.2a	335.8 ± 4.4c	843.6 ± 8.1a
DHA	101.1 ± 3.1b	104.5 ± 3.5b	99.1 ± 3.1b	107.0 ± 3.5b	571.3 ± 4.5a	103.3 ± 3.4b	565.3 ± 4.4a	103.8 ± 3.4b
AsA redox	0.888 ± 0.10a	0.861 ± 0.07a	0.889 ± 0.08a	0.880 ± 0.07a	0.368 ± 0.06b	0.890 ± 0.09a	0.373 ± 0.02b	0.890 ± 0.07a
Cysteine	7.28 ± 0.49b	6.19 ± 0.43c	7.31 ± 0.51b	6.28 ± 0.46c	57.63 ± 0.67a	6.24 ± 0.48c	53.20 ± 0.61a	6.27 ± 0.40c
H_2_S	0.063 ± 0.01c	0.059 ± 0.009c	0.065 ± 0.01c	0.061 ± 0.01c	0.059 ± 0.01c	0.148 ± 0.04b	0.061 ± 0.01c	0.248 ± 0.07a

Data are mean ± standard error of four replicates. Means followed by different lowercase letters indicate significant differences for a particular trait at *P* < 0.05 by ANOVA followed by Duncan’s Multiple Range Tests

### Cys content, endogenous H_2_S level and response of thiol-metabolizing enzymes

Compared to MuC, foliar Cys content in *pvsod1* mutant did not change significantly in any of the treatment regimes (Table 2). Cys level was close to MC value in As-treated VL-63 but doubled when BSO was co-applied with As (Table 2). The H_2_S level showed significant increase (2.5-fold) over MuC in As + BSO-treated *pvsod1* mutant and became 4.2-fold higher in the mutant at As + BSO + NaHS (Table 2). Compared to MC, change in H_2_S level was not significant in VL-63 when As was co-imposed with BSO and also with NaHS (Table 2).

Activities of OAS-TL, γ-ECS, PCS increased significantly (*P* = 0.018–0.011) over MuC in As-treated mutant (Table 3). At As + BSO, γ-ECS activity was not detectable while PCS level reduced significantly (*P* ≤ 0.0001) in both the genotypes. The OAS-TL level in the mutant was close to MuC, but LCD and DCD activity increased by about 2.5- to 2.7-fold over MuC in the mutant. Activities of all five enzymes followed trend similar to As + BSO in both the genotypes subjected to As + BSO + NaHS treatment. Change was not significant in rest of the cases (Table 3).

**Table 3:** Activity of foliar OAS-TL (nmol Cys min^−1^ mg^−1^ protein), γ-ECS (nmol γ-EC min^−1^ mg^−1^ protein), PCS (nmol GSH eq min^−1^ mg^−1^ protein), LCD (nmol H_2_S min^−1^ mg^−1^ protein), DCD (nmol H_2_S min^−1^ mg^−1^ protein), and activities of SOD (U mg^−1^ protein), APX (µmol AsA oxi min^−1^ mg^−1^ protein), DHAR (µmol AsA formed min^−1^ mg^−1^ protein), GR (nmol NADPH oxi min^−1^ mg^−1^ proten), GST (Units mg^−1^ protein), CAT (nmol H_2_O_2_ min^−1^ mg^−1^ protein) and GO (µmol glyoxylate mg^−1^ protein min^−1^) in mother genotype (*Phaseolus vulgaris* L.) VL-63 and *pvsod1* mutant exposed to 50 µM As (sodium arsenate), As + 1 mM BSO and As + BSO + 100 μM NaHS for 10 days treatment period

Traits	MC (VL-63)	MuC (*pvsod1*)	As-treated		As + BSO		As + BSO + NaHS	
			VL-63	*Pvsod1*	VL-63	*Pvsod1*	VL-63	*pvsod1*
OAS-TL	15.2 ± 0.09b	14.4 ± 0.10b	15.3 ± 0.09b	37.3 ± 0.19a	16.0 ± 0.10b	11.0 ± 0.09b	16.3 ± 0.11b	13.1 ± 0.10b
γ-ECS	0.42 ± 0.09b	0.47 ± 0.09b	0.44 ± 0.09b	0.82 ± 0.32a	0.00 ± 0.00c	0.00 ± 0.00c	0.00 ± 0.00c	0.00 ± 0.00c
PCS	0.81 ± 0.07c	0.93 ± 0.07b	0.79 ± 0.05c	1.49 ± 0.34a	0.09 ± 0.01d	0.13 ± 0.02d	0.09 ± 0.02d	0.14 ± 0.05d
LCD	21.41 ± 0.58b	19.41 ± 0.58b	20.94 ± 0.53b	17.88 ± 0.67b	21.14 ± 0.49b	44.49 ± 0.67a	20.41 ± 0.57b	43.51 ± 0.67a
DCD	18.08 ± 0.51b	16.10 ± 0.51b	19.02 ± 0.51b	17.30 ± 0.59b	18.20 ± 0.50b	36.38 ± 0.5a	18.10 ± 0.53b	35.40 ± 0.59a
SOD	41.8 ± 3.4c	14.2 ± 2.2c	121.7 ± 6.7b	14.3 ± 2.3c	148.8 ± 7.9a	15.0 ± 2.2c	143.9 ± 7.5a	14.7 ± 2.4c
APX	90.6 ± 4.3c	103.7 ± 4.2b	91.2 ± 4.8c	110.7 ± 4.6b	45.1 ± 3.9d	178.7 ± 8.2a	45.4 ± 4.0d	181.7 ± 8.5a
DHAR	0.59 ± 0.07b	0.50 ± 0.11b	0.61 ± 0.08b	0.90 ± 0.11a	0.30 ± 0.03c	0.93 ± 0.23a	0.33 ± 2.3c	0.90 ± 0.24a
GR	41.6 ± 2.2b	48.9 ± 3.6b	42.3 ± 2.8b	88.9 ± 3.6a	43.0 ± 2.7b	46.9 ± 4.6b	42.5 ± 2.5b	48.9 ± 4.9b
GST	0.15 ± 0.04c	0.26 ± 0.09b	0.10 ± 0.04c	1.03 ± 0.08a	0.05 ± 0.00d	0.11 ± 0.04c	0.06 ± 0.01d	0.13 ± 0.04c
CAT	51.4 ± 5.7b	47.4 ± 6.2b	47.7 ± 4.3b	31.4 ± 6.2c	49.4 ± 5.0b	69.3 ± 6.0a	49.8 ± 4.4b	70.1 ± 6.1a
GO	0.81 ± 0.03b	0.78 ± 0.03b	0.83 ± 0.04b	0.78 ± 0.03b	1.67 ± 0.09a	1.83 ± 0.09a	1.77 ± 0.09a	1.78 ± 0.03a

Data are mean ± standard error of four replicates. Means followed by different lowercase letters indicate significant differences for a particular trait at *P* < 0.05 by ANOVA followed by Duncan’s Multiple Range Tests

### Response of antioxidant enzymes and photorespiratory ROS production

Foliar SOD activity was constitutively low in *pvsod1* mutant but increased significantly over MC in VL-63 exposed to three treatment protocols. Barring CAT activity which assayed low, activities of APX, DHAR, CAT and GR increased significantly (*P* ≤ 0.010) in the mutant exposed to As only (Table 3). At As + BSO and As + BSO + NaHS, activities of APX and DHAR were reduced by about twofold to fourfold in VL-63 but along with CAT, increased significantly (*P* ≤ 0.002) in the mutant (Table 3). GST activity increased over MuC by about fourfold in the As-treated mutant but reduced significantly below MuC level when BSO was co-applied in the medium. In As-treated VL-63, GST level reduced by about twofold of MC value and further declined in As + BSO and As + BSO + NaHS medium (Table 3). Significant (*P* = 0.0017) increase was observed in activity of GO, a major source of photorespiratory H_2_O_2_ production, in both VL-63 and *pvsod1* mutant exposed to As + BSO and at NaHS + As + BSO (Table 3). Enzyme activity did not change significantly in rest of the cases.

### Changes in foliar H_2_O_2_ content, lipid peroxidation and electrolyte leakage %

Foliar H_2_O_2_ content, MDA and EL % were significantly higher (*P* ≤ 0.001) in VL-63 than MC at 50 µM As and further increased at As + BSO and maintained the level at As + BSO + NaHS (Table 4). Compared to MuC, changes were non-significant (*P* = 0.151) in *pvsod1* mutant throughout the treatment regimes (Table 4).

**Table 4:** Foliar H_2_O_2_ (µmol g^−1^ FW), malondealdehyde (MDA, nmol g^−1^ FW) and electrolyte leakage (EL %) in mother control genotype VL-63 and *pvsod1* mutant in 50 µM As (sodium arsenate), As + 1 mM BSO and As + BSO + 100 μM NaHS for 10 days treatment period

Traits	MC (VL-63)	MuC (*pvsod1*)	As-treated		As + BSO		As + BSO + NaHS	
			VL-63	*Pvsod1*	VL-63	*Pvsod1*	VL-63	*pvsod1*
H_2_O_2_	4.4 ± 0.7b	4.7 ± 0.8b	22.3 ± 1.0a	4.9 ± 0.8b	18.8 ± 1.1a	5.1 ± 0.8b	18.9 ± 0.9a	3.9 ± 0.8b
MDA	4.1 ± 0.7 b	5.2 ± 0.9 b	18.7 ± 1.0a	4.3 ± 0.2b	16.9 ± 1.0a	4.9 ± 0.7b	21.6 ± 1.1a	4.1 ± 0.2b
EL %	3.3 ± 0.5b	3.4 ± 0.6b	19.1 ± 1.2a	4.1 ± 0.3b	20.8 ± 1.2a	5.5 ± 0.8a	20.3 ± 1.2a	4.4 ± 0.5b

Data are mean ± standard error of four replicates. Means followed by different lowercase letters indicate significant differences for a particular trait at* P* < 0.05 by ANOVA followed by Duncan’s Multiple Range Tests

### Gene expression pattern of selected genes

**Fig. 2:**
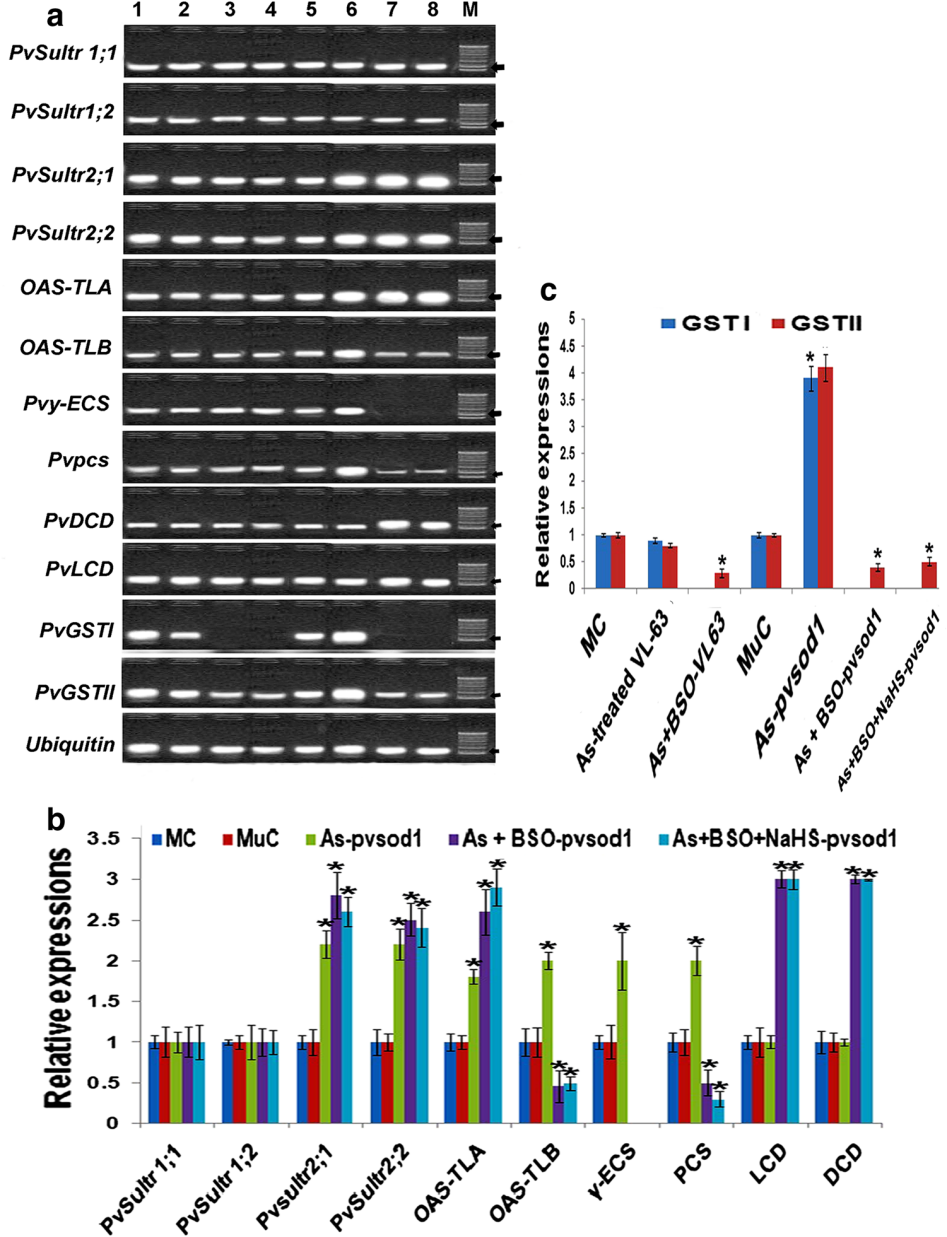
Transcript analysis of four sulfate transporters, OAS-TL, γ-ECS, PCS, DCD, LCD, and GSTs isoforms in leaves (leaflets + petioles) of VL-63 and *pvsod1* mutant of common bean, under control (0 μM of As), As (Sodium arsenate, 50 μM)-treated, As + 1 mM BSO, and As + 1 mM BSO + 100 μM NaHS treatments by **a** qRT-PCR, followed by 2 % agarose gel electrophoresis with *ubiquitin* used for cDNA normalization and **b**, **c** their relative expression levels. Data are mean ± SE of three biological replicates with average of three technical replicates/biological replicate. *Asterisk* denotes the significant changes (up- or down-regulation) in relation to control (set as 1) at *P* < *0.05*. *Lane 1* mother control, *2* As-treated VL-63, *3* As + BSO-treated VL-63, *4* As + BSO + NaHS-treated VL-63, *5* mutant control, *6* As-treated mutant, *7* As + BSO-treated mutant, *8* As + BSO + NaHS-treated mutant; M-100-bp DNA marker (M) (*arrow* 200 bp)

**Fig. 3:**
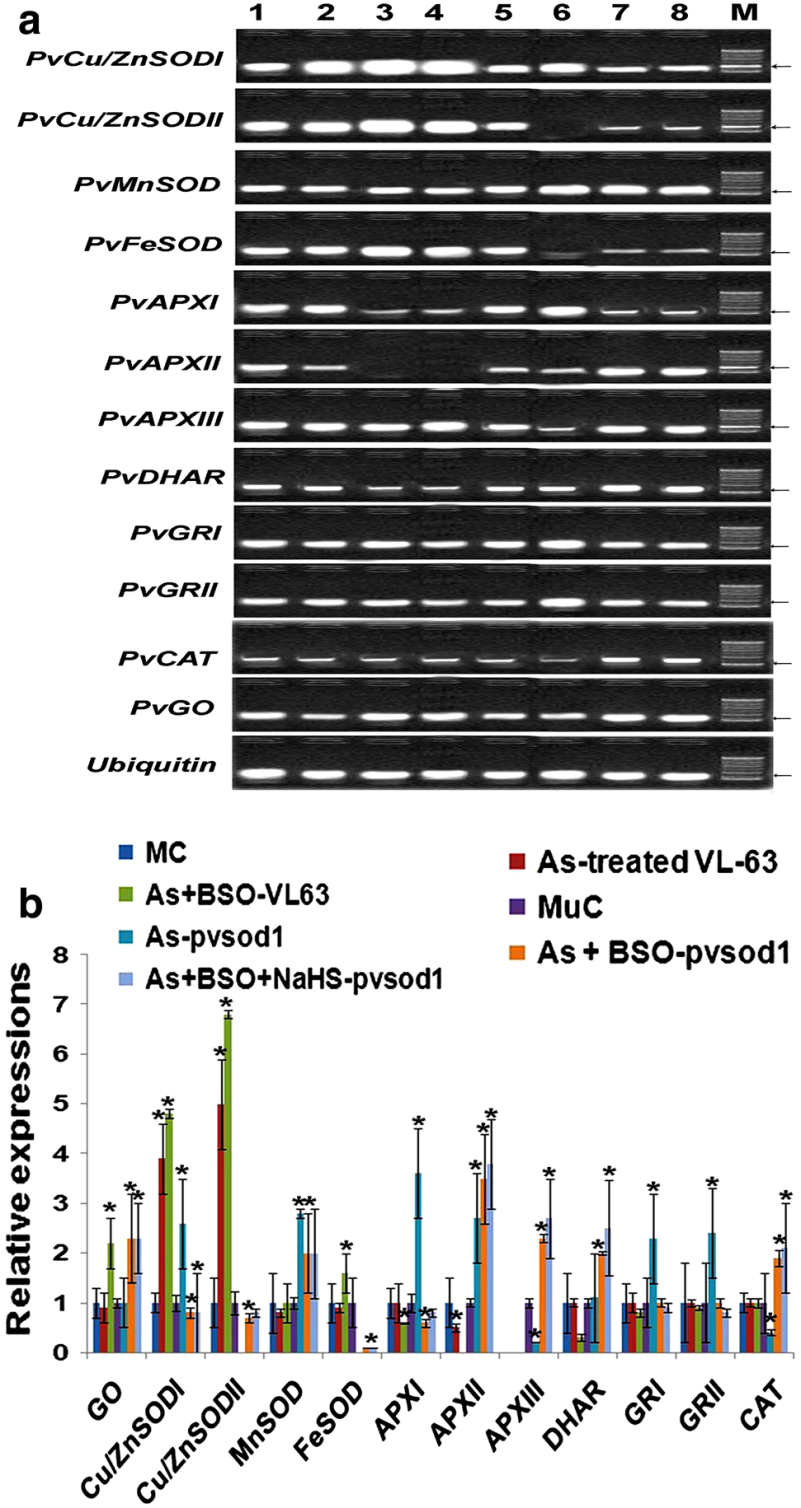
Transcript analysis of Cu/Zn SOD, Mn SOD, Fe SOD, APX, DHAR, GR, CAT, and GO isoforms in leaves (leaflets + petioles) of VL-63 and *pvsod1* mutant of common bean, under control (0 μM of As), As (Sodium arsenate, 50 μM)-treated, As +1 mM BSO, and As + 1 mM BSO + 100 μM NaHS treatment protocols by qRT-PCR, followed by 2 % agarose gel electrophoresis with *ubiquitin* used for cDNA normalization (**a**) and (**b**) their relative expression levels. Data are mean ± SE of three biological replicates with average of three technical replicates/biological replicate. *Asterisk* denotes the significant changes (up- or down-regulation) in relation to control (set as 1) at *P* < *0.05*. *Lane 1* Mother control, *2* As-treated VL-63, *3* As + BSO-treated VL-63, *4* As + BSO + NaHS-treated VL-63, *5* Mutant control, *6* As-treated mutant, *7* As + BSO-treated mutant, *8* As + BSO + NaHS-treated mutant; M-100-bp DNA marker (M) (*arrow* 200 bp)

At 50 µM As, *PvSultr2;1* and *PvSultr* *2;2* sulfate transporters were up-regulated by about 2.2-fold in *pvsod1* mutant while *PvSultr 1;1* and *PvSultr 1;2* changed non-significantly (*P* = 0.351) in both the genotypes (Fig. 2a, b). In As-treated *pvsod1* mutant, *OAS*-*TL A* and *OAS*-*TL B* were induced by about 1.8- to 2-fold, while γ-ECS and PCS were up-regulated over MuC by about twofold (Fig. 2a, b). LCD, DCD and GO transcripts were as per control level in *pvsod1* mutant. Transcripts of *APX I*, *APX II*, *APX III*, *DHAR*, *GRI* and *II*, and *GST I* and *II* were significantly (*P* = 0.003) up-regulated in the mutant but CAT repressed (Figs. 2a–c, 3a, b). Except *Mn SOD* and *Cu/Zn SODI* which was induced, transcripts other SOD isoforms were not detectable in As-treated mutant (Fig. 3a, b). Barring significant up-regulation of *Cu/Zn SOD I* and *II*, other changes were not significant (*P* = 0.56) in VL-63 under As treatment alone.

At As + BSO, *PvSultr 2;1*, *PvSultr 2;2*, and *OAS*-*TLA* isoforms were up-regulated by about 2.8-, 2.5- and 2.9-fold, respectively, while *OAS*-*TL B* was down-regulated in the mutant. Transcripts of *LCD* and *DCD* were induced by nearly threefold in *pvsod1* mutant whereas GO transcript was elevated in both the genotypes by about 2.3-fold (Fig. 2a, b). Expressions of γ-ECS transcript could not be detected while PCS expression was down-regulated in both the genotypes (Fig. 2a, b). Among antioxidant defense components, expressions of SOD transcripts particularly *Cu/Zn SOD I* and *II* were significantly (*P* ≤ 0.0001) elevated in VL-63 but remained low in *pvsod1* mutant. Expressions of *APX II* could not be detected whereas DHAR was significantly down-regulated and change was not significant for GR and CAT expressions in VL-63. *GST II* repressed and *GST I* transcript was not expressed in both the genotypes in presence of BSO in the medium (Fig. 2a, c). In *pvsod1* mutant, apart from *APX I* and *II*, a third isoform *APX III* expressed along with more than twofold up-regulations of CAT and DHAR transcripts over MuC (Fig. 3a, b). The *APX III* isoform although down-regulated during As treatment alone, was up-regulated by about 2.3- and 2.7-fold over MuC in presence of BSO (Fig. 3b).

At As + BSO + NaHS, expressions of different transcripts followed more or less same trend in both the genotypes in relation to As + BSO treatment (Figs. 2, 3).

## Discussion

Present common bean genotypes exhibited contrasting responses to 50 µM As treatment. Shoots and roots dry masses were not affected in *pvsod1* mutant but shoot growth was severely inhibited in VL-63. This is despite the lower shoot: root As ratio (<1.0) in VL-63 but higher (>1.0) in the mutant. This apparently conflicting situation can be better explained if we take the response of both genotypes in As + BSO and As + BSO + NaHS treatments under consideration. BSO is a specific inhibitor of GSH biosynthesis and in absence of BSO, available GSH pool can facilitate GSH-As binding, thus preventing the translocation of As from the root to shoot in VL-63. The reverse trend in accumulation pattern i.e. higher shoot to root (>1.0) in VL-63 under BSO exposure was presumably due to the absence of enough GSH pool, which perturbed the sequestration of As in roots, resulting into maximum translocation of As (V) to the shoots. In this backdrop, high As accumulation and normal growth (MuC like) of the shoots indicated greater As tolerance and localized detoxification capability of the mutant compared with that found in VL-63.

Growing evidences indicate critical importance of S in As tolerance and detoxification, and GSH plays central role in this process (Finnegan and Chen 2012; Talukdar 2013). During its synthesis, GSH exclusively requires Cys as one of its building blocks, and thus stimulation in up-stream thiol cascade is required to keep the Cys and GSH levels as per the cellular requirements. In the *pvsod1* mutant, this stimulation was possible due to enhanced expressions of sulfate transporters and activity of OAS-TL, γ-ECS and PCS. Significant up-regulation of *PvSultr2;1* and *PvSultr2;2* in leaves of As-treated *pvsod1* mutant strongly indicated induction of sulfate transporters involved in xylem loading of sulfate and its subsequent transport from root to shoot to meet the growing S demand in photosynthetic organs due to overaccumulation of As. Significant increase in OAS-TL, γ-ECS and PCS activities in the mutant might be due to elevated expressions of *OAS*-*TL A and B*, γ-ECS and PCS transcripts. Stimulation of this entire thiol cascade machinery indicated that high As-exposure necessitated greater thiol demand in tolerant genotype which is being met through coordinated induction of thiol cascade. Obviously, normal level (close to control) of Cys in As-treated *pvsod1* mutant is a strong indication of availability of enough thiol pools to meet the escalating consumption of downstream thiol moieties.

Proper augmentation of thiolic capacity with antioxidant defense components is essential in conferring tolerance to As stress. Enhanced GR activity due to >twofold up-regulation of both *GRI* and *GRII* in As-treated *pvsod1* mutant ensured effective recycling of GSH by preventing excess build-up of GSSG. Increased GSTs activity in the As-treated mutant was mainly due to threefold to fourfold induction of *GSTI* and *II* transcripts. GR transcript was found elevated in As-treated Indian mustard (Khan et al. 2009) while GST activity and its transcripts were up-regulated in *Arabidopsis*, rice, lentils and *Brassica* subjected to As treatment (Abercrombie et al. 2008; Chakrabarty et al. 2009; Srivastava et al. 2009; Talukdar and Talukdar 2014a). Besides GR and GSTs, enhanced transcriptional expressions of *APX I and II* isoforms led to As-induced elevation of APX activity in the *pvsod1* mutant. Significant increase in SOD activity in VL-63 was orchestrated through elevated expressions of *Cu/Zn SOD I* and *Cu/Zn SOD II* transcripts, indicating As-induced excess superoxide generation. The minimum SOD level in *pvsod1* mutant was maintained by enhanced expressions of *Cu/Zn SODI* and *MnSOD* isoforms. The result pointed out that cytosolic isoforms (Cu/Zn SODs) in both bean genotypes played pivotal roles in maintaining SOD activity during As-exposures, which confirmed earlier findings (Abercrombie et al. 2008; Talukdar and Talukdar 2013, 2014a). Obviously, stimulated SOD activity in the present case ensured effective dismutation of As-induced excess superoxide generation but at the same time it produced H_2_O_2_ as bi-product. Increased APX and GST levels, supported by DHAR and GR, have not only ensured effective scavenging of H_2_O_2_ and lipid peroxides but also maintained favorable AsA as well as GSH redox (>0.8) in the As-treated mutant despite down-regulation of CAT. Considerable differences were found between VL-63 and *pvsod1* mutant for GSH level even in the absence of As which might be due to their genotypic differences and might not be directly influenced by SOD activity. This fact was further substantiated by the significant increase of GSH in the mutant over VL-63 under As-exposure alone but SOD activity was consistently low in the mutant irrespective of treatment protocols. The direct effect of SOD in controlling the level of GSH in the present case thus seems unclear and needs further study. Absence of any induction in up-stream thiol metabolisms and reduction in downstream ROS-scavenging capacity led to excess H_2_O_2_ generation in photosynthetic organs of VL-63. This triggered elevated level of lipid peroxidation and membrane leakage, marking the onset of As-induced oxidative stress in the mother genotype.

Functional interplay between Cys metabolisms and down-steam antioxidant defense was more evidenced when BSO was co-applied with As and also with As + NaHS. The *pvsod1* seedlings effectively counterbalanced As-induced oxidative stress by managing H_2_O_2_ and lipid peroxidation level within control level in presence of BSO despite complete inhibition of γ-ECS activity, nearly fivefold decrease in GSH redox and significant down-regulation of PCS transcripts. Furthermore, absence of *GST I* and repression of *GST II* isoforms led to sharp decline in GST activity in As-treated mutant, indicating reduction in GSH-dependent antioxidant defense capability due to non-availability of enough GSH pool. In this scenario, significant induction of GO transcripts suggested a strong linkage between inhibition of GSH biosynthesis and photorespiratory H_2_O_2_ generation capacity of the plant. Inverse relationship between GSH pool and expressions of GO transcripts was also observed in *rlfL*-*1* mutant of grass pea in which diminishing GSH redox due to BSO treatment led to enhanced GO expressions and photorespiratory H_2_O_2_ production (Talukdar and Talukdar 2014b). In this backdrop, increasing AsA content coupled with enhanced activity of DHAR, APX and CAT suggested induction in AsA-mediated antioxidant defense in the *pvsod1* mutant as a bypass mode of GSH. Interestingly, *APX III* isoform was unique in the mutant which was up-regulated only when BSO was added in the medium. It is also noteworthy that apart from *APX I*, expressions of all the isoforms of APX, DHAR and CAT elevated markedly in this treatment compared to As treatment alone.

Remarkably enough, Cys level in the *pvsod1* mutant did not change significantly in relation to its MuC, despite blockage of its channeling to GSH by BSO. Accumulated free Cys, but not GSH, has the capacity to act as a prooxidant within cell and can affect cellular redox balance (Park and Imlay 2003). Perhaps, Cys level in the present mutant was managed by inducing its degradation through desulfuration and by regulating its synthesis through OAS-TL. LCD and DCD play predominant roles in Cys desulfuration which is pivotal in Cys-degraded H_2_S generation within cell (Bloem et al. 2004; Chen et al. 2011). Substantial enhancement in LCD/DCD transcripts and their activity in *pvsod1* mutant in presence of BSO strongly indicated metabolic diversion of Cys through its degradation, thus, preventing its build up at prooxidant level and indicating that Cys-degradation pathway is induced when its utilization to GSH synthesis is inhibited (here by BSO). Mutants deficient in Cys-desulfuration pathway exhibited overaccumulation of free Cys which led to excess ROS generation and oxidative imbalance (Álvarez et al. 2010; Talukdar 2014) but relieved when Cys-desulfuration was induced (Talukdar and Talukdar 2014b). H_2_S treatment has been implicated in inducing GSH-mediated plant stress tolerance (Calderwood and Kopriva 2014) but it is not known whether GSH is the sole receiver of the H_2_S effects. Cys desulfuration in the present As + BSO- and As + BSO + NaHS-treated *pvsod1* mutant was accompanied with the huge accumulation of endogenous H_2_S and stimulation of AsA-dependent antioxidant defense. High H_2_S has the capacity to regulate OAS-TL activity through Cys-synthase complex, triggering association of complex and decrease in OAS-TL activity (Hell and Wirtz 2011). In the *pvsod1* mutant, OAS-TL activity was regulated by counterbalancing up-regulation of *OAS*-*TLA* with down-regulation of its *OAS*-*TLB* isoform in presence of BSO. The result strongly confirmed metabolic diversion of Cys to H_2_S through induction of desulfuration pathway. Obviously, along with induced desulfuration, the up-stream regulation of Cys synthesis was necessitated to prevent excess build-up of free Cys in view of a blockage of its downstream channeling to GSH. It is thus apparent that fates of up-stream thiol status depend on functional interplay between downstream thiol-components, preferably between GSH and H_2_S both of which require Cys to be built-up. This huge rise in H_2_S level in the *pvsod1* mutant could be compared as ‘H_2_S burst’, which coupled with significant enhancement of APX, DHAR and CAT activity and decline in GO level resulted in significant reduction in ROS-induced oxidative damage in the mutant. Certainly, GSH is not the sole receiver of H_2_S effects. In the present study, H_2_S seems to play dual roles; maintained and stabled Cys level and induced AsA-dependent antioxidant defense in *pvsod1* mutant against As-toxicity as an alternate mode of GSH-dependent defense. In agreement with the present findings, BSO-treated *rlfL*-*1* mutant of *Lathyrus* induced Cys desulfuration which played prominent roles in reversal of cell proliferations and restoration of normal mitosis during low GSH level (Talukdar and Talukdar 2014b). GSH synthesis was also blocked in VL-63 but there was no induction in H_2_S production in the genotype. This possibly led to lowering of AsA redox and AsA-dependent antioxidant defense under As-exposure. Obviously, low H_2_S level in the mother genotype could not functionally compensate the loss of GSH pool in the combined presence of As and BSO.

## Conclusions

Present study revealed functional interplay between Cys-generated H_2_S at up-stream and GSH-dependent antioxidant defense in downstream thiol metabolisms in presence of As (V). Results indicated onset of As-induced oxidative stress in VL-63 due to lack of responsiveness of its entire thiol cascade. Contrastingly, *pvsod1* mutant exhibited As tolerance even when BSO was co-applied with As. The mutant induced Cys-degradation pathway to generate huge endogenous H_2_S which stimulated AsA-mediated antioxidant defense and regulated Cys synthesis via OAS-TL in the background of low GSH redox and effectively prevented As-induced oxidative stress. The study pointed out that H_2_S holds the key in cellular Cys homeostasis and modulation of antioxidant defense against As-toxicity. Also, a metabolic diversion is imminent when Cys-consumption route towards GSH is blocked and GSH may not be the sole receiver of endogenous H_2_S-mediated cellular signaling during As tolerance of plants.
